# Predictors of lead break during transvenous lead extraction

**DOI:** 10.1002/joa3.12524

**Published:** 2021-03-15

**Authors:** Junji Morita, Kyohei Yamaji, Michio Nagashima, Yusuke Kondo, Yohei Sadohara, Jun Hirokami, Rei Kuji, Kengo Korai, Masato Fukunaga, Kenichi Hiroshima, Kenji Ando, Masahiko Goya

**Affiliations:** ^1^ Department of Cardiology Kokura Memorial Hospital Kitakyushu Japan; ^2^ Department of Cardiovascular Medicine Chiba University Graduate School of Medicine Chiba Japan; ^3^ Department of Cardiovascular Medicine Tokyo Medical and Dental University Tokyo Japan

**Keywords:** coradial lead, lead break, pacemaker lead, passive lead, transvenous lead extraction

## Abstract

**Background:**

The incidence, predictors, and clinical impact of lead break during transvenous lead extraction (TLE) were previously unknown.

**Methods:**

We included consecutive patients who underwent TLE between September 2013 and July 2019 at our institute. Lead break during removal was defined as lead stretching and becoming misshapen, as assessed by fluoroscopy.

**Results:**

A total of 246 patients underwent TLE for 501 leads. At a patient level, complete success was achieved in 226 patients (91.9%). At a lead level, 481 leads (96.0%) were completely removed and 101 leads (20.1%) were broken during the procedure. Of 392 identified pacemaker leads, 71 (18.3%) were broken during the TLE procedure. A multivariable analysis confirmed high lead age (odds ratio [OR] 1.12, 95% confidence interval (CI) 1.07‐1.17; *P* < .001), passive leads (OR 2.29 95% CI 1.09‐4.80; *P* = .028), coradial leads (OR 3.45 95% CI 1.72‐6.92; *P* < .001), and insulators made of nonpolyurethane (OR 2.38 95% CI 1.03‐5.26; *P* = .04) as predictors of lead break. Broken leads needed longer procedure times and were associated with a higher rate of cardiac tamponade.

**Conclusions:**

Lead age, coradial bipolar leads, passive leads, and leads without polyurethane insulation were predictors of lead break and could increase the difficulty of lead extraction.

## Graphical abstract

The extraction of cardiac implantable electronic device leads due to infections, malfunctions, and recalls is common, and can lead to complications such as lead break resulting in incomplete lead removal and cardiac tamponade. We found that older lead age, co‐radial leads, passive leads, and non‐polyurethane leads were independent predictors of lead break during extraction. These findings will guide clinicians to make appropriate choices of procedure and take greater care when these risk factors are present.

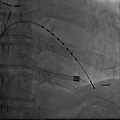

## 1 INTRODUCTION

Cardiac implantable electronic devices (CIEDs) are widely used for the treatment of electrophysiological disorders, being implanted in 1.2‐1.4 million patients per year.^1^, ^2^, ^3^ Accordingly, the number of lead explants or extractions owing to infections caused by CIEDs, lead malfunctions, and recalls has increased up to 30 000 per year.^3^, ^4^

Compared with surgical lead removal, lead removal using a transvenous approach is less invasive and associated with lower risk of complications.^5^ However, transvenous lead extraction (TLE), defined as transvenous removal of leads which have been in place for longer than a year or complicated leads requiring the assistance of specialized equipment, is often complicated by lead break followed by incomplete lead removal and/or further complications such as cardiac tamponade. While passive leads and older leads are reported to be at high risk of lead break,^6^ the impact on break during TLE of detailed lead structure such as insulator, coil, and lead size remained unknown.

We therefore conducted a single‐center observational registry study of lead removal including detailed information on lead structure and procedure outcomes.

## 2 METHODS

### 2.1 Patients population

We recruited consecutive patients who underwent either surgical or transvenous lead removal between September 2013 and July 2019 at our institute. Of these patients, we included those who underwent TLE, and excluded those who underwent lead explant (removal of leads within 1 year of implantation by manual traction) and those who underwent lead removal for a subcutaneous implantable cardioverter defibrillator (S‐ICD). TLE was defined as any TLE in which at least one lead required the assistance of equipment not typically required during implantation or at least one lead had been in place for longer than 1 year.^3^

### 2.2 Extraction procedure and lead break

**FIGURE 1:**
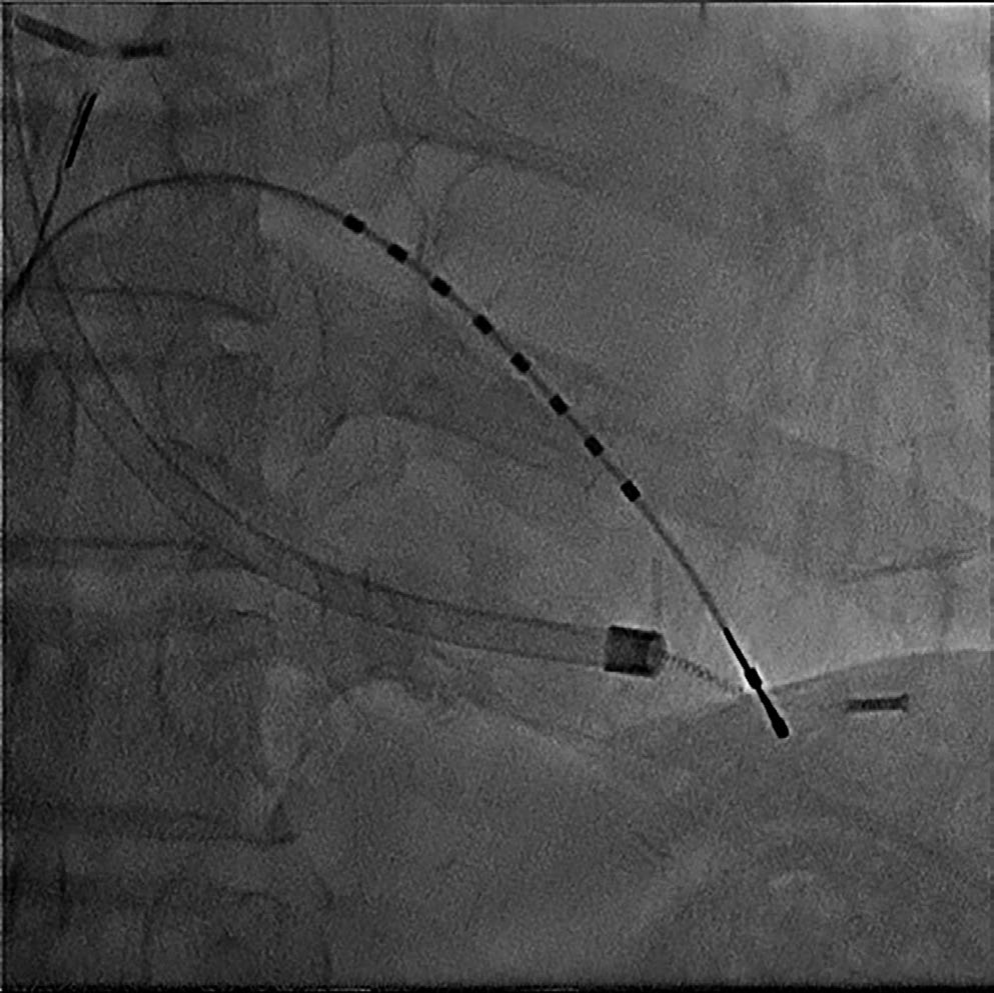
Lead break during transvenous lead extraction as assessed by fluoroscopy

The procedures were performed under general or intravenous anesthesia according to the patient's condition. TLE was performed as previously described, using a variety of approaches and tools including simple manual traction, locking stylets, laser sheaths, femoral snares, mechanical sheaths, and rotational mechanical sheaths.^3^, ^7^, ^8^ Minimum traction with a regular pacemaker stylet was applied to all leads at the beginning of each case. If manual traction did not result in successful lead extraction, an SLS II Excimer Laser Sheath (Spectranetics) with a locking stylet was normally used. Our method of using a laser sheath required two operators. One operator pushed the laser sheath while the other operator pulled the locking stylet. All extraction procedures were performed by one of the two experienced operators: operator A and B had an experience of performing TLE for 3 and 4 years, respectively. In some instances, different techniques and tools were used at the discretion of the operating physician, including an Evolution Mechanical Dilator Sheath (Cook Medical), adapted mechanical sheaths, and snares using a femoral approach. Lead break during removal was defined as the lead stretching and becoming misshapen, as assessed by fluoroscopy (Figure 1).

### 2.3 Clinical outcomes

Outcomes of TLE were defined in accordance with the 2017 HRS consensus statement and 2018 European Heart Rhythm Association (EHRA) expert consensus statement.^3^, ^7^ Complete success was defined as the complete extraction of all the targeted leads from the body of the patient with the absence of any complication or procedure‐related death. Partial success was defined as removal of all targeted leads with the retention of no more than a small portion (<4 cm) of lead without any complication or procedure‐related death. Failure was defined as the inability to achieve either complete or partial success. Complete lead removal was defined as the successful removal of all targeted lead material. Partial lead removal was defined as retention of a small part (<4 cm) of the lead and incomplete lead removal as a result of retention of the lead part ≥4 cm.

### 2.4 Statistical analysis

Categorical variables are presented as number and percentage and were compared using the chi‐square test. Continuous variables are expressed as mean ± SD or median with interquartile range and were compared using the Student *t* test or the Wilcoxon rank‐sum test depending on their distributions. Multivariable logistic regression analysis was used to identify risk factors for lead break. Odds ratios (OR) and their 95% confidence interval (95% CI) were reported. All analyses were performed with JMP software version 13.2.1 (SAS Institute Inc). All reported P values were two‐tailed, and *P* < .05 were considered statistically significant.

## 3 RESULTS

### 3.1 Clinical outcomes

During the study period, a total of 265 patients with 532 leads underwent lead removal. Of those, we excluded 1 patient (2 leads) who underwent elective open heart surgery, 2 patients (2 leads) with S‐ICD, and 16 patients (27 leads) with leads placed within 1 year and removed by manual traction (ie, lead explant). Ultimately, 246 patients who underwent TLE for 501 leads were included (Figure 1). The patient characteristics are summarized in Table 1. TLE was indicated because of infection in 204 patients (83%) (pocket infection: 156 patients [63%]; endocarditis: 48 patients [20%]), abandoned lead in 38 patients (15%), thrombosis/vascular issues in 8 patients (3%), recalled lead in 3 patients (1%), lead perforation in 2 patients (1%), to facilitate access for magnetic resonance imaging in 2 patients (1%), and tricuspid regurgitation caused by a malapposed lead in 1 patient (0.4%). The implanted device was a pacemaker in 166 patients (67%), an ICD in 49 patients (20%), cardiac resynchronized therapy and defibrillator in 27 patients (11%), and cardiac resynchronized therapy and pacemaker in 4 patients (2%).

**TABLE 1:** Baseline characteristics of patients who underwent transvenous lead extraction

	All patients (n = 246)	Patients with at least one lead break (n = 76)	Patients without lead break (n = 170)	*P* value
Age, y	72 ± 16	73 ± 16	69 ± 16	.13
Male gender	181 (74)	60 (79)	121 (71)	.20
Height, cm	160 ± 10	162 ± 11	159 ± 10	.047
Body weight, kg	57 ± 14	60 ± 13	56 ± 14	.07
Hypertension	148 (60)	45 (59)	103 (61)	.84
Diabetes	62 (25)	17 (22)	45 (26)	.49
Coronary artery disease	45 (18)	9 (12)	36 (21)	.08
Valvular disease	36 (15)	11 (14)	25 (15)	1.0
Hemodialysis	15 (6)	3 (4)	12 (7)	.40
Atrioventricular block	81 (33)	21 (28)	60 (35)	.24
Sick sinus syndrome	74 (29)	22 (29)	52 (31)	.07
Left ventricular ejection fraction, %	55 ± 13	54 ± 14	56 ± 13	.33
Pacemaker	166 (67)	46 (61)	120 (71)	.12
CRT‐P	4 (2)	1 (1)	3 (2)	1.0
ICD	49 (20)	19 (25)	30 (18)	.18
CRT‐D	27 (11)	10 (13)	17 (10)	.46
Number of extracted leads				
1	67 (27)	15 (20)	52 (31)	.08
2	123 (50)	37 (49)	86 (51)	.78
3	39 (16)	16 (21)	23 (14)	.14
4	13 (5)	7 (9)	6 (4)	.07
5	4 (2)	1 (1)	3 (2)	1.0
Infection	204 (83)	70 (92)	134 (79)	.01
Pocket infection	156 (63)	54 (71)	102 (60)	.1
Sepsis/endocarditis/bacteremia	48 (20)	16 (21)	32 (19)	.68
Noninfection	42 (17)	6 (8)	36 (21)	.01
Abandoned lead	38 (15)	6 (8)	32 (19)	.03
Thrombosis/Vascular issues	8 (3)	1 (1)	7 (4)	.44
Recalled lead	3 (1)	0 (0)	3 (2)	.55
Lead perforation	2 (1)	0 (0)	2 (1)	1.0
Tricuspid regurgitation	1 (0.4)	0 (0)	1 (0.6)	1.0
Facilitate access to MRI	2 (1)	0 (0)	2 (1)	1.0

Abbreviations: CRT‐P, cardiac resynchronization therapy pacemaker; ICD, implantable cardioverter defibrillator; CRT‐D, cardiac resynchronization therapy defibrillator; MRI, magnetic resonance imaging.

**FIGURE 2:**
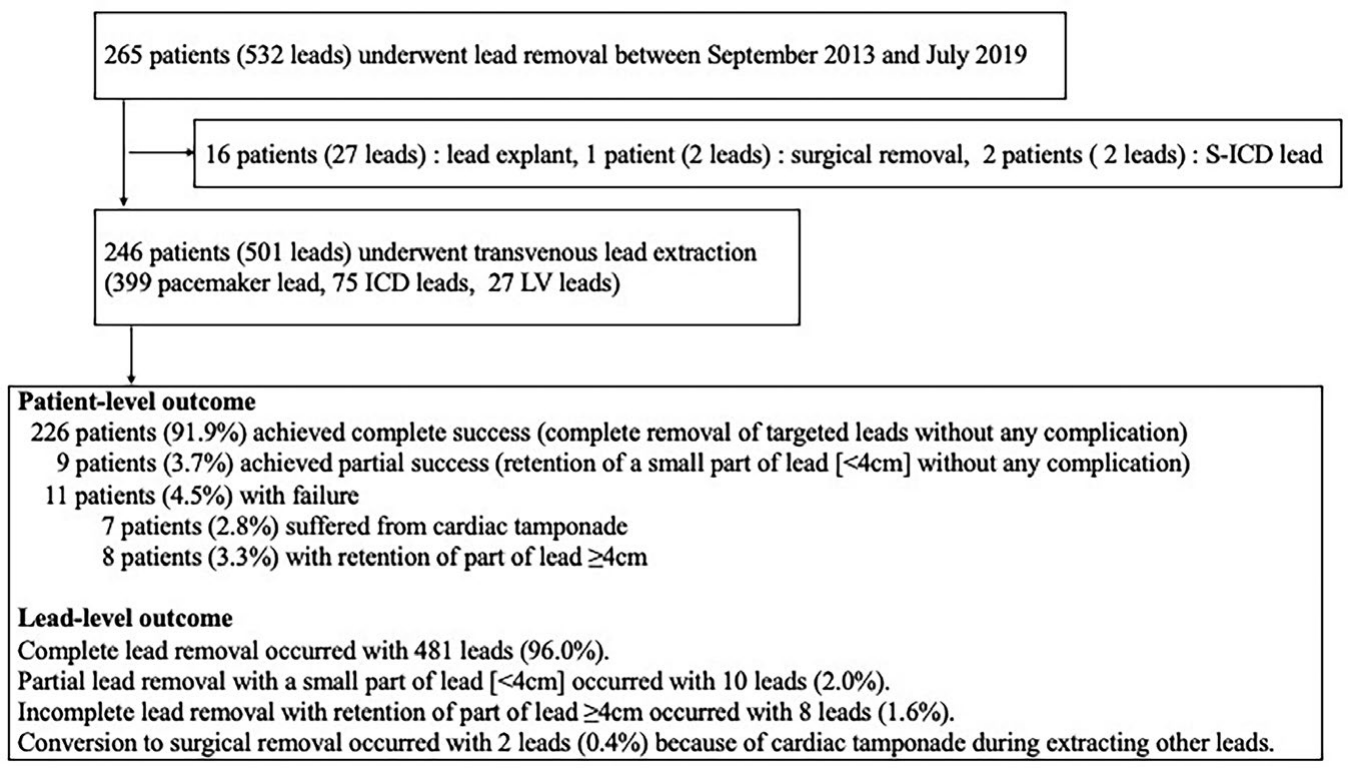
Study flowchart

Regarding patient‐level outcome, complete success was achieved in 226 patients (91.9%) while procedure failure occurred in a total of 11 patients (4.5%) (Figure 2). Cardiac tamponade occurred in 7 patients (2.8%), of whom 2 patients with 7 leads required surgical repair. Retention of part of lead (≥4 cm) occurred in a total of 8 patients (3.3%).

Regarding lead‐level outcome, 481 leads were completely removed (96.0%) and 101 leads (20.1%) were broken during the procedure. Of the 101 broken leads, partial lead removal (retention of <4 cm lead) occurred in 10 leads and incomplete lead removal (retention of ≥4 cm lead) occurred in 8 leads. There were no differences in the complete success rate (90.4% vs 92.1%, *P* =.66) or complication rate (3.8% vs 2.6%, *P* =.34) between operators A and B.

### 3.2 Nonbroken versus Broken leads

After excluding 75 ICD leads, 27 LV leads, 2 leads which needed to be removed surgically, and 5 unknown pacemaker leads, we divided 392 pacemaker leads into 2 groups: 318 nonbroken (81.1%) and 74 broken (18.9%) leads. The characteristics of these pacemaker leads are summarized in Table 2. The mean lead age was 9.4 ± 6.6 years. There were 212 passive leads (54.1%) and 180 active leads (46.0%), and 264 coaxial leads (68.2%) and 119 coradial leads (30.4%). A total of 186 leads (47.5%) had insulators made of only silicon and 84 leads (21.4%) were made of only polyurethane.

**TABLE 2:** Baseline lead characteristics according to broken leads

	All leads (n = 392)	Break (n = 74)	No break (n = 318)	*P* value
Lead age, years	9.4 ± 6.6	15.5 ± 0.7	7.8 ± 0.4	<.001
Number of leads extracted	2.4 ± 0.9	2.5 ± 1.0	2.4 ± 0.9	.38
Indication				
Infection	360 (91.8)	71 (96.0)	289 (90.9)	.24
Pocket infection	262 (66.8)	50 (67.6)	212 (66.7)	.88
Sepsis/endocarditis/bacteremia	98 (25.0)	21 (28.4)	77 (24.2)	.46
Vegetation	54 (13.8)	13 (17.6)	41 (12.9)	.29
Noninfection	32 (8.2)	3 (4.1)	29 (9.1)	.24
Abandoned lead	29 (7.4)	3 (4.1)	26 (8.2)	.32
Thrombosis/Vascular issues	9 (2.3)	1 (1.4)	8 (2.5)	1.0
Lead perforation	2 (0.5)	0 (0)	2 (0.63)	1.0
Tricuspid regurgitation	1 (0.26)	0	1 (0.31)	1.0
Facilitate access to MRI	1 (0.26)	0	1 (0.31)	1.0
Lead type				
Passive lead	212 (54.1)	62 (83.8)	150 (47.2)	<.001
Active lead	180 (46.0)	12 (16.2)	168 (52.8)	<.001
Bipolar lead	388 (99.0)	71 (96.0)	317 (99.7)	.02
Co‐axial lead	264 (68.2)	35 (49.3)	229 (72.5)	<.001
Co‐radial lead	119 (30.4)	33 (44.6)	86 (27.0)	.003
Lead body size, mm	1.99 ± 0.3	2.0 ± 0.3	1.99 ± 0.3	.76
Silicon insulator	186 (47.5)	30 (40.4)	156 (49.1)	.19
Polyurethane insulator	84 (21.4)	9 (12.2)	75 (23.6)	.03
Polyurethane and ETFE insulator	65 (16.6)	17 (23.0)	48 (15.1)	.06
Silicon and optim insulator	29 (7.4)	10 (13.5)	19 (6.0)	.07
Silicon and polyurethane insulator	20 (5.1)	6 (8.1)	14 (4.4)	.14
Other insulator	8 (2.0)	2 (2.7)	6 (1.9)	.65
Location				
Right atrium	196 (50.0)	38 (51.4)	158 (49.7)	.80
Right ventricle	196 (50.0)	36 (48.7)	160 (50.3)	.80

Abbreviations: ETFE, ethylene tetrafluoroethylene; MRI, magnetic resonance imaging.

The multivariable analyses revealed that older lead age (OR 1.12, 95% CI 1.08‐1.17, *P* <.001), passive leads (OR 2.36, 95% CI 1.13‐4.91, *P* =.02), coradial leads (OR 3.33, 95% CI 1.67‐6.65; *P* <.001), and insulators made of nonpolyurethane (OR 2.46, 95% CI 1.08‐5.62; *P* =.03) were independent predictors of lead break (Table 3). Extraction tools were more often used for broken leads (100% vs 72.6%, *P* <.001). Broken leads were associated with a longer procedure time (from insertion of locking stylet to extraction: 25.1 ± 41.2 minutes vs 5.2 ± 11.6 minutes, *P* <.001) and a higher rate of cardiac tamponade (14.9% vs 1.6%, *P* <.001) (Table 4). Table S1 includes the results of lead breaks for each lead product. ThinLine/Fineline (passive; Boston Scientific) had the highest lead break rate of all lead products (52.9%).

**TABLE 3:** Univariate and multivariate analysis of predictors of lead breaks during transvenous lead extraction

Variables	Univariable		Multivariable	
	OR (95% CI)	*P* value	OR (95% CI)	*P* value
Lead age, per 1‐y increase	1.13 (1.09‐1.17)	<.001	1.12 (1.08‐1.17)	<.001
Vegetation	1.33 (0.79‐2.26)	.29	1.56 (0.69‐3.54)	.28
Passive lead	4.39 (2.44‐7.88)	<.001	2.36 (1.13‐4.91)	.02
Co‐radial lead	1.85 (1.23‐2.77)	.003	3.33 (1.67‐6.65)	<.001
Nonpolyurethane lead	1.97 (1.02‐3.79)	.03	2.46 (1.08‐5.62)	.03
Lead body size, per 1 mm increase	1.16 (0.45‐2.98)	.76	1.12 (0.37‐3.47)	.84
Number of leads extracted	1.13 (0.86‐1.48)	.38	1.02 (0.74‐1.40)	.83

Abbreviations: CI, confidence interval; OR, odds ratio.

**TABLE 4:** Device type, procedure time, and procedure outcomes of transvenous lead extraction

	All leads (n = 392)	Break (n = 74)	No break (n = 318)	*P* value
Simple traction with standard or locking stylet	87 (22.2)	0 (0)	87 (27.4)	<.001
Laser	288 (73.4)	72 (97.3)	216 (68.0)	<.001
Mechanical sheath	22 (5.6)	14 (18.3)	8 (2.5)	<.001
Snare	28 (7.1)	17 (23.0)	11 (3.2)	<.001
Rotational mechanical sheath	16 (4.1)	4 (5.4)	12 (3.8)	.51
Procedure time per lead, minutes	9.4 ± 21.5	25.1 ± 41.2	5.2 ± 11.6	<.001
Cardiac tamponade	16 (4.1)	11 (14.9)	5 (1.6)	<.001
Complete lead removal	374 (95.4)	56 (75.7)	318 (100)	<.001

### 3.3 Risk factors of cardiac tamponade or fragment retention of broken leads

Of the 74 broken leads, cardiac tamponade or fragment retention occurred in 25 leads. The characteristics of the broken leads are summarized in Table 5. The mean age of leads was 15.2 ± 7.2 years. There were 62 passive leads (83.8%) and 38 coaxial leads (51.4%). A total of 33 leads (44.6%) had insulators without polyurethane. There were no significant differences in the lead characteristics between with and without cardiac tamponade and retained fragment. Among broken leads, univariate analysis showed that there were no significant differences between the 2 groups (Table 6). Table S2 includes the results of broken leads with retained fragments or cardiac tamponade for each lead product. The complication rates of CapSure Z (passive; Medtronic), ThinLine/Fineline (passive; Boston Scientific), ThinLine/Fineline II (passive; Boston Scientific), and ThinLine/Fineline (active) were 100% (1/1), 33.3% (3/9), 83.3% (5/6), and 20% (1/5), respectively.

**TABLE 5:** Baseline lead characteristics according to broken leads with cardiac tamponade or retained fragments

	Broken lead (n = 74)	Cardiac tamponade or lead retention (n = 25)	Without cardiac tamponade or lead retention (n = 49)	*P* value
Lead age, y	15.2 ± 7.2	15.6 ± 7.6	15.0 ± 7.0	.72
Number of leads extracted	1.8 ± 0.9	1.8 ± 1.0	1.8 ± 0.8	.84
Indication				
Infection	71 (96.0)	24 (96.0)	47 (96.0)	1.0
Pocket infection	50 (67.6)	16 (64.0)	34 (69.4)	.64
Sepsis/endocarditis/bacteremia	21 (28.4)	8 (32.0)	13 (26.5)	.62
Vegetation	13 (17.6)	5 (20.0)	8 (16.3)	.7
Lead type				
Passive lead	62 (83.8)	22 (88.0)	40 (81.6)	.74
Active lead	12 (16.2)	3 (12.0)	9 (18.4)	.74
Co‐axial lead	38 (51.4)	9 (36.0)	29 (59.2)	.06
Co‐radial lead	33 (44.6)	14 (56.0)	19 (38.8)	.16
Lead body size, mm	2.0 ± 03	2.0 ± 0.3	2.0 ± 0.3	.24
Silicon insulator	30 (40.5)	8 (32.0)	22 (44.9)	.29
Polyurethane insulator	9 (12.2)	5 (20.0)	4 (8.2)	.16
Polyurethane and ETFE insulator	21 (28.4)	9 (36.0)	12 (24.5)	.3
Silicon and polyurethane insulator	11 (14.9)	3 (12.0)	8 (6.3)	.74
Nonpolyurethane lead	33 (44.6)	8 (32.0)	25 (51.0)	.12

Abbreviation: ETFE, ethylene tetrafluoroethylene.

**TABLE 6:** Univariate analysis of predictors of cardiac tamponade or retained fragments among broken leads

Variables	Univariable	
	OR (95% CI)	*P* value
Lead age > 20 y	1.51 (0.55‐4.13)	.42
Passive lead	1.65 (0.40‐6.73)	.49
Co‐radial lead	2.01 (0.76‐5.34)	.15
Nonpolyurethane lead	2.21 (0.81‐6.08)	.11
Vegetation	1.28 (0.37‐4.42)	.69

Abbreviations: CI, confidence interval; OR, odds ratio.

## 4 DISCUSSION

This single‐center observational study had the following salient findings. First, the incidence of pacemaker leads break during TLE was 18.9%. Second, older lead age, coradial leads, passive leads, and nonpolyurethane leads were independently associated with lead break. Third, 24.3% of lead break resulted in incomplete lead removal, and lead break had long procedure times and occasionally resulted in cardiac tamponade.

### 4.1 Structure and materials of lead break

Pacing leads have major components: electrodes, conductors, insulation, and fixation mechanism, which could cause break owing to strong stress. Lead break that occurred during TLE was defined as lead stretching and becoming misshapen, as assessed by fluoroscopy. The introduction of powered sheaths including laser sheaths has facilitated the removal of leads with a high age.^9^, ^10^, ^11^ de Bie et al reported that the incidence of lead break was 14.7% in 251 patients who underwent manual traction or use of snares to remove leads with an age of 4.2 years.^6^ However, the rate of lead break using a powered sheath was unknown. In our study, 77.8% of leads were removed using a powered sheath and the mean lead age was 9.4 ± 6.6 years. Lead break occurred in 29.2% of passive leads, and passive leads were an independent predictor of lead break. Over time, fibrous tissue develops in the electrode‐myocardial interfaces of the heart, especially in the tip of the electrode.^12^, ^13^ In studies analyzing passive leads, old leads were more difficult to extract and passive leads were also easy to be broken.^6^, ^14^, ^15^, ^16^ These studies and our data suggest that passive leads develop stronger adhesion to the fibrous tissue and could be more easily broken than active leads. Lead break occurred in only 10.7% of polyurethane insulator leads. Insulators of pacing leads are generally made of polyurethane or silicon. Polyurethane leads have higher tear strength and lower friction coefficient than silicon leads.^17^ A relationship between lead insulator and TLE has rarely been reported, but one small previous study reported that polyurethane insulation was a predictor of procedure difficulty in lead removal.^18^ However, in the present study, polyurethane insulators had a low risk of break. In our opinion, the insulator is stressed during TLE and stronger polyurethane leads are less likely to be broken. Lead break occurred in 27.7% of coradial leads. There are two kind of bipolar lead design: coradial lead and coaxial lead. Coaxial leads have an outer anode coil and inner cathode coil separated with inner insulation and the lead is covered in outer insulation, whereas coradial leads have individually insulated wires wound together around the center lumen and outer insulation. Thus, there is no inner insulation or outer coil as found in the conventional coaxial bipolar pacing in coradial leads.^19^, ^20^ Although the reason for the risk of break of coradial leads is unknown, having only one layer of insulation and coil may cause weakness susceptible to the stress caused by pulling.

### 4.2 Complications of TLE

To our knowledge, the present study is the first to assess procedure outcomes in lead extraction where break occurs. Procedure time of the broken lead was significantly longer than that of the nonbroken lead. There are some possible reasons that procedure times are longer in cases of lead break. First, lead breaks require switching to a femoral approach, and therefore a longer procedure time. Second, when a lead breaks, the procedure must be gentle to avoid cutting off the lead. In the present study, the rate of cardiac tamponade of the break lead was significantly higher than that of the nonbroken lead. The adhesion between the lead and the myocardium is generally strong when lead break occurs. Removing the tip of the lead, which has strong adhesion to the myocardium, could result in the surrounding tissue to tear off. This is the hypothesis that the risk of cardiac tamponade is high when lead break occurs. The result of this study suggests that lead type should be considered during implantation of a new pacemaker. We should be careful of the risks of lead break and consider a femoral approach and gentle extraction if leads being extracted are old or passive, coradial, or without polyurethane. Furthermore, there were no significant risks of complications related to TLE in this study. However, surgical removal might be considered instead of TLE if coradial or nonpolyurethane leads are being extracted to prevent complications, such as retained fragments and cardiac tamponade.

### 4.3 Limitations

The present study had some limitations, being a retrospective analysis in a single center. First, a larger study population and multiple centers are necessary to further validate the current findings. Second, the method of TLE was determined at the discretion of the operating physician. Third, the usage rate of the rotational mechanical sheath was low at 4.1% because it was not approved for regular use in Japan until September 2018. Fourth, lead break was defined by fluoroscopy, and it is possible that a few cases of partial lead break were not detected by this method. The clinical result is applicable to fluoroscopy‐apparent lead break. Last, unknown leads and leads which required conversion to sternotomy were excluded from this study. This is unlikely to have an impact on the results, as the number of excluded lead was small.

## 5 CONCLUSIONS

In TLE, break occurred in 18.3% pacemaker leads. Lead age, coradial bipolar leads, passive leads, and leads without polyurethane insulation were predictive of pacemaker lead break and could increase difficulty of lead extraction.

When leads being extracted are old or passive, coradial, or without polyurethane, we should be careful of the risks of lead break and consider a femoral approach and gentle extraction.

## CONFLICTS OF INTEREST

Authors declare no Conflict of Interests for this article.

## INFORMED CONSENT

The institutional review board (IRB) at our center approved the trial, and all patients provided written informed consent before participating. Date of IRB approval is March 16, 2016, and the IRB number is 16031603.

## Supporting information

Table S1

Table S2

## References

[1] Greenspon AJ , Patel JD , Lau E , Ochoa JA , Frisch DR , Ho RT , et al. Trends in permanent pacemaker implantation in the United States from 1993 to 2009: increasing complexity of patients and procedures. J Am Coll Cardiol. 2012;60:1540–5. 22999727 10.1016/j.jacc.2012.07.017

[2] Raatikainen MJ , Arnar DO , Merkely B , Camm AJ , Hindricks G . Access to and clinical use of cardiac implantable electronic devices and interventional electrophysiological procedures in the European Society of Cardiology Countries: 2016 Report from the European Heart Rhythm Association. Europace. 2016;18(suppl 3):iii1–79. 27496955 10.1093/europace/euw244

[3] Kusumoto FM , Schoenfeld MH , Wilkoff BL , Berul CI , Birgersdotter‐Green UM , Carrillo R , et al. 2017 HRS expert consensus statement on cardiovascular implantable electronic device lead management and extraction. Heart Rhythm. 2017;14:e503–e551. 28919379 10.1016/j.hrthm.2017.09.001

[4] Hauser RG , Katsiyiannis WT , Gornick CC , Almquist AK , Kallinen LM . Deaths and cardiovascular injuries due to device‐assisted implantable cardioverter‐defibrillator and pacemaker lead extraction. Europace. 2010;12:395–401. 19946113 10.1093/europace/eup375 PMC2825385

[5] Wilkoff BL , Love CJ , Byrd CL , Bongiorni MG , Carrillo RG , Crossley GH , et al. Transvenous lead extraction: Heart Rhythm Society expert consensus on facilities, training, indications, and patient management: this document was endorsed by the American Heart Association (AHA). Heart Rhythm. 2009;6:1085–104. 19560098 10.1016/j.hrthm.2009.05.020

[6] de Bie MK , Fouad DA , Borleffs CJ , van Rees JB , Thijssen J , Trines SA , et al. Trans‐venous lead removal without the use of extraction sheaths, results of >250 removal procedures. Europace. 2012;14:112–6. 21873627 10.1093/europace/eur269

[7] Bongiorni MG , Burri H , Deharo JC , Starck C , Kennergren C , Saghy L , et al. 2018 EHRA expert consensus statement on lead extraction: recommendations on definitions, endpoints, research trial design, and data collection requirements for clinical scientific studies and registries: endorsed by APHRS/HRS/LAHRS. Europace. 2018;20:1217. 29566158 10.1093/europace/euy050

[8] Goya M , Nagashima M , Hiroshima K‐I , Hayashi K , Makihara YU , Fukunaga M , et al. Lead extractions in patients with cardiac implantable electronic device infections: single center experience. J Arrhythm. 2016;32:308–12. 27588155 10.1016/j.joa.2016.02.004 PMC4996845

[9] Wilkoff BL , Byrd CL , Love CJ , Hayes DL , Sellers TD , Schaerf R , et al. Pacemaker lead extraction with the laser sheath: results of the pacing lead extraction with the excimer sheath (PLEXES) trial. J Am Coll Cardiol. 1999;33:1671–6. 10334441 10.1016/s0735-1097(99)00074-1

[10] Bordachar P , Defaye P , Peyrouse E , Boveda S , Mokrani B , Marquié C , et al. Extraction of old pacemaker or cardioverter‐defibrillator leads by laser sheath versus femoral approach. Circ Arrhythm Electrophysiol. 2010;3:319–23. 20562442 10.1161/CIRCEP.109.933051

[11] Wazni O , Epstein LM , Carrillo RG , Love C , Adler SW , Riggio DW , et al. Lead extraction in the contemporary setting: the LExICon study: an observational retrospective study of consecutive laser lead extractions. J Am Coll Cardiol. 2010;55:579–86. 20152562 10.1016/j.jacc.2009.08.070

[12] Epstein AE , Kay GN , Plumb VJ , Dailey SM , Anderson PG . Gross and microscopic pathological changes associated with nonthoractomy implantable defibrillator leads. Circulation. 1998;98:1517–24. 9769305 10.1161/01.cir.98.15.1517

[13] Maytin M , Epstein LM , John RM . Lead implant duration does not always predict ease of extraction: extraction sheath may be required at <1 year. Pacing Clin Electrophysiol. 2011;34:1615–20. 22017453 10.1111/j.1540-8159.2011.03225.x

[14] Henrikson CA , Zhang K , Brinker JA . A survey of the practice of lead extraction in the United States. Pacing Clin Electrophysiol. 2010;33:721–6. 20132504 10.1111/j.1540-8159.2010.02692.x

[15] Bongiorni MG , Blomstrom‐Lundqvist C , Kennergren C , Dagres N , Pison L , Svendsen JH , et al. Current practice in transvenous lead extraction: a European Heart Rhythm Association EP Network Survey. Europace. 2012;14:783–6. 22622992 10.1093/europace/eus166

[16] Sohal M , Williams SE , Arujuna A , Chen Z , Bostock J , Gill JS , et al. The current practice and perception of cardiac implantable electronic device transvenous lead extraction in the UK. Europace. 2013;15:865–70. 23180646 10.1093/europace/eus383

[17] Stokes K , Cobian K . Polyether polyurethanes for implantable pacemaker leads. Biomaterials. 1982;3:225–31. 7171682 10.1016/0142-9612(82)90024-2

[18] Cecchin F , Atallah J , Walsh EP , Triedman JK , Alexander ME , Berul CI . Lead extraction in pediatric and congenital heart disease patients. Circ Arrhythm Electrophysiol. 2010;3:437–44. 20729392 10.1161/CIRCEP.110.957324

[19] Tang C , Yeung‐Lai‐Wah JA , Qi A , Mills P , Clark J , Tyers F . Initial experience with a co‐radial bipolar pacing lead. Pacing Clin Electrophysiol. 1997;20:1800–7. 9249835 10.1111/j.1540-8159.1997.tb03570.x

[20] Fahraeus T , Israel CW , Wollenstein M . Thin co‐radial bipolar leads: technology and clinical performance. Herzschrittmacherther Elektrophysiol. 2001;12:148–57. 27432334 10.1007/s003990170019

