# Supplementary material for: Predictors of lead break during transvenous lead extraction
Source: J Arrhythm. 2021 Mar 15;37(3):645–52. doi: 10.1002/joa3.12524 (PMC8207345; doi:10.1002/joa3.12524)
Supplement: Supplementary file 1 — Table S1 [file JOA3-37-645-s001.docx]

**Table S1. Lead breaks for each lead product**

|  | **All leads** | **Break** | **No break** | **p value** |
| --- | --- | --- | --- | --- |
| Isoflex S (passive) | 23 | 5 (21.7) | 8 (78.3) | 0.78 |
| Isoflex Optim (passive) | 7 | 0 (0) | 7 (100) | 0.36 |
| OptiSense Optim (active) | 5 | 0 (0) | 5 (100) | 0.59 |
| Tendril SDX (active) | 29 | 1 (3.5) | 28 (96.5) | 0.03 |
| Target-Tip (passive) | 4 | 0 (0) | 4 (100) | 1.0 |
| CapSure SP (passive) | 9 | 0 (0) | 9 (100) | 0.22 |
| CapSure Sence (passive) | 1 | 0 (0) | 1 (100) | 1.0 |
| CapSure SP Novus (passive) | 3 | 2 (66.7) | 1 (33.3) | 0.09 |
| CapSure Z (passive) | 1 | 1 (100) | 0 (0) | 0.19 |
| CapSure Z Novus (passive) | 28 | 2 (7.1) | 26 (92.9) | 0.13 |
| CapSureFix (active) | 10 | 0 (0) | 10 (100) | 0.22 |
| CapSureFix Novus (active) | 32 | 1 (3.1) | 31 (96.9) | 0.02 |
| CapSureFix Novus MRI (active) | 0 | 0 (0) | 12 (100) | 0.13 |
| CapSure VDD-2 (passive) | 8 | 3 (37.5) | 5 (62.5) | 0.18 |
| SelectSecure (active) | 1 | 0 (0) | 1 (100) | 1.0 |
| Excellence (passive) | 6 | 1 (16.7) | 5 (83.3) | 1.0 |
| ThinLine/Fineline (passive) | 17 | 9 (52.9) | 8 (47.1) | 0.001 |
| ThinLine/Fineline EZ (active) | 1 | 0 (0) | 1 (100) | 1.0 |
| ThinLine/Fineline Ⅱ (passive) | 23 | 6 (26.1) | 17 (73.9) | 0.41 |
| ThinLine/Fineline Ⅱ EZ (active) | 18 | 5 (27.8) | 13 (72.2) | 0.35 |
| Screwvine (active) | 17 | 0 (0) | 17 (100) | 0.05 |
| Petite (passive) | 6 | 1 (16.7) | 5 (83.3) | 1.0 |
| Petite (active) | 1 | 0 (0) | 1 (100) | 1.0 |
| Beflex (active) | 7 | 0 (0) | 7 (100) | 0.36 |
| DEXTRUS (active) | 2 | 0 (0) | 2 (100) | 1.0 |
| INGEVITY (active) | 11 | 0 (0) | 11 (100) | 0.23 |
| INGEVITY (passive) | 2 | 0 (0) | 2 (100) | 1.0 |
| Retrox (active) | 3 | 1 (33.3) | 2 (66.7) | 0.47 |
| Synox (passive) | 2 | 0 (0) | 2 (100) | 1.0 |
| Setrox (active) | 1 | 0 (0) | 1 (100) | 1.0 |
| Sielo (active) | 5 | 0 (0) | 5 (100) | 0.59 |
| Solia (active) | 5 | 0 (0) | 5 (100) | 0.59 |
|  | | | | |
