# Supplementary material for: Predictors of lead break during transvenous lead extraction
Source: J Arrhythm. 2021 Mar 15;37(3):645–52. doi: 10.1002/joa3.12524 (PMC8207345; doi:10.1002/joa3.12524)
Supplement: Supplementary file 2 — Table S2 [file JOA3-37-645-s002.docx]

**Table S2. Lead breaks with cardiac tamponade or lead retention for each lead product**

|  | **Broken leads** | **Cardiac tamponade or lead retention** | **Without cardiac tamponade or lead retention** | **p value** |
| --- | --- | --- | --- | --- |
| Isoflex S (passive) | 5 | 0 (0) | 5 (100) | 0.16 |
| Tendril SDX (active) | 1 | 0 (0) | 1 (100) | 1.0 |
| CapSure SP Novus (passive) | 2 | 0 (0) | 2 (100) | 0.55 |
| CapSure Z (passive) | 1 | 1 (100) | 0 (0) | 0.34 |
| CapSure Z Novus (passive) | 2 | 0 (0) | 2 (100) | 0.55 |
| CapSureFix Novus (active) | 1 | 0 (0) | 1 (100) | 1.0 |
| CapSure VDD-2 (passive) | 3 | 0 (0) | 3 (100) | 0.55 |
| Excellence (passive) | 1 | 0 (0) | 1 (100) | 1.0 |
| ThinLine/Fineline (passive) | 9 | 3 (33.3) | 6 (66.7) | 1.0 |
| ThinLine/Fineline Ⅱ (passive) | 6 | 5 (83.3) | 1 (16.7) | 0.02 |
| ThinLine/Fineline Ⅱ EZ (active) | 5 | 1 (20) | 4 (80) | 0.66 |
| Petite (passive) | 1 | 0 (0) | 1 (100) | 1.0 |
| Retrox (active) | 1 | 0 (0) | 1 (100) | 1.0 |
|  | | | | |
